# Free Will and Neuroscience: From Explaining Freedom Away to New Ways of Operationalizing and Measuring It

**DOI:** 10.3389/fnhum.2016.00262

**Published:** 2016-06-01

**Authors:** Andrea Lavazza

**Affiliations:** Neuroethics, Centro Universitario InternazionaleArezzo, Italy

**Keywords:** readiness potential, unconscious decision, choice prediction, stochastic processes, measurement of freedom, evidence accumulation

## Abstract

The concept of free will is hard to define, but crucial to both individual and social life. For centuries people have wondered how freedom is possible in a world ruled by physical determinism; however, reflections on free will have been confined to philosophy until half a century ago, when the topic was also addressed by neuroscience. The first relevant, and now well-known, strand of research on the brain correlates of free will was that pioneered by Libet et al. (1983), which focused on the allegedly unconscious intentions taking place in decisions regarded as free and voluntary. Libet’s interpretation of the so-called readiness potential (RP) seems to favor a sort of deflation of freedom (Soon et al., 2008). However, recent studies seem to point to a different interpretation of the RP, namely that the apparent build-up of the brain activity preceding subjectively spontaneous voluntary movements (SVM) may reflect the ebb and flow of the background neuronal noise, which is triggered by many factors (Schurger et al., 2016). This interpretation seems to bridge the gap between the neuroscientific perspective on free will and the intuitive, commonsensical view of it (Roskies, 2010b), but many problems remain to be solved and other theoretical paths can be hypothesized. The article therefore, proposes to start from an operationalizable concept of free will (Lavazza and Inglese, 2015) to find a connection between higher order descriptions (useful for practical life) and neural bases. This new way to conceptualize free will should be linked to the idea of “capacity”: that is, the availability of a repertoire of general skills that can be manifested and used without moment by moment conscious control. The capacity index, which is also able to take into account the differences of time scales in decisions, includes reasons-responsiveness and is related to internal control, understood as the agent’s ownership of the mechanisms that trigger the relevant behavior. Cognitive abilities, needed for one to have capacity, might be firstly operationalized as a set of neuropsychological tests, which can be used to operationalize and measure specific executive functions, as they are strongly linked to the concept of control. Subsequently, a free will index would allow for the search of the underlying neural correlates of the capacity exhibited by people and the limits in capacity exhibited by each individual.

## Introduction—Free Will as a Problem (Not Only) for Science

The concept of free will is hard to define, but crucial to both individual and social life (Kane, 2005). Free will can be the reason why someone is not sent to jail during a trial upon appealing to insanity: the subject was not “free” when they committed the crime, not because someone was pointing a gun to their head, but because a psychiatric illness prevented them from controlling their actions. According to a long-standing philosophical tradition, if someone was not “free” when they did something, they cannot be held responsible for their deed (Glannon, 2015). And the freedom in question is both “social” freedom (linked to constraints imposed by our peers or by external factors), and the one indicated by the term *free will*.

Free will can be defined by three conditions (Walter, 2001). The first one is the “ability to do otherwise.” This is an intuitive concept: to be free, one has to have at least two alternatives or courses of action between which to choose. If one has an involuntary spasm of the mouth, for example, one is not in the position to choose whether to twist one’s mouth or not. The second condition is the “control over one’s choices.” The person who acts must be the same who decides what to do. To be granted free will, one must be the author of one’s choices, without the interference of people and of mechanisms outside of one’s reach. This is what we call agency, that is, being and feeling like the “owner” of one’s decisions and actions. The third condition is the “responsiveness to reasons”: a decision can’t be free if it is the effect of a random choice, but it must be rationally motivated. If I roll a dice to decide whom to marry, my choice cannot be said to be free, even though I will freely choose to say “I do”. On the contrary, if I choose to marry a specific person for their ideas and my deep love for them, then my decision will be free.

Thus defined, free will is a kind of freedom that we are willing to attribute to all human beings as a default condition. Of course there are exceptions: people suffering from mental illness and people under psychotropic substances (Levy, 2013). Nevertheless, the attribution of free will as a general trend does not imply that all decisions are always taken in full freedom, as outlined by the three conditions illustrated above: “We often act on impulse, against our interests, without being fully aware of what we are doing. But this does not imply that we are not potentially able to act freely. Ethics and law have incorporated these notions, adopting the belief that usually people are free to act or not to act in a certain way and that, as a result, they are responsible for what they do, with the exceptions mentioned above” (Lavazza and Inglese, 2015).

It is commonly experienced that the conditions of “ability to do otherwise”, “control” and “responsiveness to reasons” are very rarely at work all at once. Moreover, they would require further discussion, because there is wide disagreement on those conditions as regards their definition and scope (Kane, 2016). But for the purposes of this article, this introductory treatment should suffice. In fact, the description of free will that I have sketched here is the one that dominated the theoretical discourse on, and practical applications of, the evaluation of human actions. From a philosophical point of view, however, starting with Plato, the main problem has been that of the actual existence of freedom, beyond the appearances and the insights that guide our daily life. The main challenge to free will has been determinism: the view that everything that happens (human decisions and actions included) is the consequence of sufficient conditions for its occurrence (Berofsky, 2011). More specifically, “It is the argument that all mental phenomena and actions are also, directly or indirectly, causally produced—according to the laws of nature (such as those of physics and neurobiology)—by previous events that lie beyond the control of the agents” (Lavazza and Inglese, 2015). Determinism was first a philosophical position; then, the birth of Galilean science—founded on the existence of immutable laws that are empirically verifiable—has increased its strength, giving rise to the concept of incompatibilism, namely the idea that free will and natural determinism cannot coexist. Only one of them can be true.

Throughout the centuries, despite its conceptual progress, philosophy hasn’t been able to solve this dilemma. As a result, today there are different irreconcilable positions about human free will: determinism is not absolute and free will exists; free will does not exist for a number of reasons, first of all (but not only) determinism; free will can exist even if determinism is true (Kane, 2011). A little more than 30 years ago, neuroscience and empirical psychology came into play. Although biological processes cannot be considered strictly deterministic on the observable level of brain functioning (nerve signal transmission), new methods of investigation of the brain, more and more precise, have established that the cerebral base is a necessary condition of behavior and even of mental phenomena. On the basis of these acquisitions, neuroscience has begun to provide experimental contributions to the debate on free will.

In order to better understand the neural bases of free will, provided that there are any, in this article I’ll review and integrate findings from studies in different fields (philosophy, cognitive neuroscience, experimental and clinical psychology, neuropsychology). Unlike previous reviews on free will and neuroscience (Haggard, 2008, 2009; Passingham et al., 2010; Roskies, 2010a; Brass et al., 2013), I have no claim of being exhaustive. My goal is to highlight a paradigm shift in the analysis and interpretation of the brain determinants preceding and/or causing free or voluntary action (Haggard, 2008 takes voluntary decision to be non-stimulus driven, as much as possible). Firstly, following Libet’s experiments, a widespread interpretation of the so-called readiness potential (RP) went in the direction of a deflation of freedom (Crick, 1994; Greene and Cohen, 2004; Cashmore, 2010; Harris, 2012). Indeed, the discovery of the role of the RP has been taken as evidence of the fact that free will is an illusion, since it seems that specific brain areas activate before we are aware of the onset of the movement. However, recent studies seem to point to a different interpretation of the RP, namely that the apparent build-up of the brain activity preceding subjectively spontaneous voluntary movements (SVM) may reflect the ebb and flow of the background neuronal noise, which is triggered by many factors (Schurger et al., 2016). This interpretation seems to bridge, at least partially, the gap between the neuroscientific perspective on free will and the intuitive, commonsensical view of it (Roskies, 2010b), but many problems remain to be solved and other theoretical paths can be hypothesized. After analyzing the change of paradigm of these perspectives, I’ll propose to start from an operationalizable concept of free will (Lavazza and Inglese, 2015) to find a connection between higher order descriptions (useful for practical life) and neural bases.

## Neuroscience: Purporting to Explain Free Will

### The Discovery of the Readiness Potential

As a preliminary consideration, it is important to underline that the idea of using an experiment (or a series of experiments) to establish whether the human being can be said to have free will implies accepting a direct link between a measurement of brain functioning and a pre-existing theoretical construct. This direct connection, as it is known, presents several problems and as we shall see, needs conceptual refinement to avoid simplifications and unfounded claims. What one can see and measure in brain activity may in fact only grasp a part of the idea of free will that we would like to test. This was one of the main criticisms to the experiments conducted so far (Mele, 2009; Nachev and Hacker, 2014). What is measured at the level of brain functioning in the laboratory does not match the concept of free will we refer to, for example, to determine whether someone who engaged in violent behavior could have done otherwise in that specific circumstance.

The first relevant, and now well-known, strand of research on the brain correlates of free will was that pioneered by Libet et al. (1983), which focused on the allegedly unconscious intentions affecting decisions regarded as free and voluntary. It should be noted that the concepts involved—“conscious intentions”, “voluntary decisions”, “free decisions”—have no clear and shared definition (Nachev and Hacker, 2014), and the experiments themselves have been differently interpreted and often criticized (Lavazza and De Caro, 2010). In any case, Libet’s experiments and their variants have been repeated several times until very recently, confirming their findings with a sufficient degree of reliability.

Libet based his work on Kornhuber and Deecke’s (1965) discovery of the *bereitschaftpotential*: the RP, a slow build-up of a scalp electrical potential (of a few microvolts), mainly measured through electroencephalography (EEG), that precedes the onset of subjectively SVM (Kornhuber and Deecke, 1965). According to its discoverers, the RP is “the electro-physiological sign of planning, preparation, and initiation of volitional acts” (Kornhuber and Deecke, 1990). “The neurobiologist John Eccles speculated that the subject must become conscious of the *intention* to act before the onset of this RP. Libet had the idea that he should test Eccles’s prediction” (Doyle, 2011).

In his experiments, Libet invited the participants to move their right wrist and to report the precise moment when they had the impression that they decided to do so, thanks to a big clock they had in front of them (Libet et al., 1983). In this way, it was possible to estimate the time of awareness with respect to the beginning of the movement, measured using an electromyogram (which records the muscle contraction). During the execution of the task, brain electrical activity was recorded through electrodes placed on the participants’ scalps. The attention was focused on a specific negative brain potential, namely the RP, originated from the supplementary motor area (SMA): a brain area involved in motor preparation, which is visible in the EEG signal as a wave that starts before any voluntary movement, while being absent or reduced before involuntary and automatic movements.

When one compares the subjective “time” of decision and what appeared at a cerebral level, the result appears as a striking blow to the traditional view of free will (Libet, 1985, 2004). In the experiment, the RP culminating in the execution of the movement starts in the prefrontal motor areas long before the time when the subject seems to have made the decision: participants became aware of their intention to take action about 350 ms after the onset of such potential. The volitional process is detected to start unconsciously 550 ms before the action is made in the case of non-preplanned acts and 1000 ms before in the case of preplanned acts. Thus these findings seem to show that our simple actions (and therefore, potentially, also more complex ones) are triggered by unconscious neural activity and that the awareness of those actions only occurs at a later time, when we think we are willing to act.

In the first phase of its intervention in the debate on free will, therefore, neuroscience seemed to argue for a deflation of freedom. Neuroscientists identified a specific aspect of the notion of freedom (the *conscious* control of the start of the action) and researched it: the experimental results seemed to indicate that there is no such conscious control, hence the conclusion that free will does not exist. However, it is important to highlight that this interpretation strongly depends on the idea that free choices or actions are fully internally generated, in the sense that they are not externally determined—where “external” means outside the subject’s conscience and the subject is something akin to the self. As we shall see, though, this distinction seems to be neither relevant nor truly informative when considering if and how choices are free.

In fact, Libet left the subject some time to veto: about 150 ms. This is the time needed for the muscles to flex in response to the command of the primary motor cortex (M2) through the spinal motor nerve cells. In the last 50 ms the action is realized with its external manifestations (bending the wrist) without any more possible intervention by the prefrontal brain areas (see “The Veto Power” Section). Libet thought there was a role for conscious will precisely in this situation: conscious will can let the action go to completion or it can block it with the explicit veto of the movement implemented by the prefrontal areas (Doyle, 2011). But the intentional inhibition of an action (a decision itself) is preceded by neural activity as well (Filevich et al., 2012, 2013). So it cannot be a completely different decision from that to take a positive decision to act.

In their experiments, Haggard and Eimer (1999) used Libet’s method, but asked the participants to perform a different task. They had to move at will either the right index finger or the left in a series of repeated trials. The authors have compared the RP and the lateralized readiness potential (LRP) in trials in which awareness appeared in shorter or longer time, that is, considering the latency of awareness compared to the RP. In their words, “the RP tended to occur later on trials with early awareness of movement initiation than on trials with late awareness, ruling out the RP as a cause of our awareness of movement initiation. However, the LRP occurred significantly earlier on trials with early awareness than on trials with late awareness, suggesting that the processes underlying the LRP may cause our awareness of movement initiation” (Haggard and Eimer, 1999). From this, one can deduce that the awareness of the intention to move one finger or the other comes after the decision was “taken by the brain”, as reflected in the LRP.

Sirigu et al. (2004) and Desmurget et al. (2009) have shown that, repeating Libet’s experiments on patients with parietal lesions, it appears that they become aware of their decision to take action only when the action itself is being carried out. In these subjects the awareness of the decision does not even come before the beginning of the movement, as it tends to coincide with the motor action. It seems that in such cases the brain alteration has reduced, if not cancelled altogether, the interval of consciousness preceding the actual implementation of the action. The authors proposed that when a movement is planned, activity in the parietal cortex, as part of a cortical sensorimotor processing loop, generates a predictive internal model of the upcoming movement. And this model might form the neural correlate of motor awareness.

Fried et al. (2011) recorded the activity of 1019 neurons as 12 subjects performed self-initiated finger movements. They found progressive neuronal recruitment, particularly in the SMA, over 1500 ms before subjects reported making the decision to move. A population of 256 SMA neurons was sufficient to predict in single trials the impending decision to move: 700 ms before the participants became aware of the decision, the accuracy of the prevision was higher than 80%. Fried et al. (2011) were also able to predict, “with a precision of a few 100 ms, the time point of that voluntary decision to move”, and they implemented a computational model thanks to which “volition emerged when a change in the internally generated firing rate of neuronal assemblies crossed a certain threshold”.

### Unreliability of the Conscious Intention

A slightly different trend of research compared to Libet’s comprises studies suggesting that the conscious intention of an action is strongly influenced by events that occur *after* the action itself was performed. In this sense, intentions are therefore partially *reconstructed* according to a process of inference, based on elements that come after the action. For instance, a study by Lau et al. (2006) has produced results that empirically support this hypothesis. The authors have used transcranic magnetic stimulation (TMS) on the pre-supplementary motor (pre-SM) area, while the subjects were performing Libet’s task. The stimulation of the pre-SM through TMS happened at different time intervals, in relation to a simple voluntary movement. When the stimulation was applied 200 ms *after* the movement, the judgment W was moved back in time, indicating that the perception of the intention was influenced by the neural activity of the pre-SM after the motor action was made (cf. also Lau et al., 2004; Lau and Passingham, 2007).

In another experiment, Banks and Isham (2009) have set a slightly different version of Libet’s task: participants were asked to push a button whenever they wanted, and *later* they had to indicate the precise moment when they had the intention to do so. When they pushed the button, subject received an auditory feedback with a delay from 5 to 60 ms, so as to give them the impression that the response happened *after* they pushed the button. Even though the subjects weren’t aware of the delay between the action and the auditory feedback, the intention to press the button was reported as happening later in time, according to a linear function with the delay of the auditory signal feedback. The identification of the moment in which the subject had intended to press the button—measured by judgment W—was therefore largely determined by the *apparent* time of the subject’s response, and not the actual answer. This result indicates that the people evaluate the time when they have had the intention to take an action based on the consequences of their action and not just on the motor action itself.

Kühn and Brass (2009) conducted an experiment combining the paradigm of the stop signal (Logan et al., 1984) with an intentional action paradigm. The subjects had to react in the quickest possible way by pushing a button as soon as a stimulus (e. g., a letter) was displayed at the center of a computer screen. Sometimes, just after the presentation of the stimulus, either a stop signal or a decision signal was shown: in the first case, the subjects had to try to stop responding; in the second case they could decide whether to press the button or stop responding. In the decision trials in which subjects had provided an answer, the subjects were asked if it had actually been the result of a decision, or if it had been inhibited—that is, if they had not been able to stop before the decision signal was presented.

The results have shown that in some instances, the subjects judged as intentional responses—i.e., as the result of a decision—those answers that in reality, on the basis of reaction times, were failed inhibitions. In other words, sometimes the subjects had a subjective experience of having intentionally decided to perform an action that they had actually not decided to take. These studies have empirically supported the hypothesis that the intentions to take voluntary actions are strongly influenced by events occurring *after* the execution of the action. In addition, they seem to confirm that the brain motor system produces a movement as the final result of its inputs and outputs; consciousness would be “informed” of the fact that a movement is going to occur and this would produce the subjective perception that the movement was decided voluntarily (Hallett, 2007).

### Predicting Choices

More recently, studying the activity of the frontal and parietal cortex, other neuroscientists of the group coordinated by Soon et al. (2008, 2013) have managed to detect the “rise” of a behavioral or abstract choice/decision (to move either the right finger or the left one; to perform a mathematical operation or another with two numbers) a few seconds before the subject becomes aware of it. An unconscious brain process has already “decided” what to do when the subject still does not know what she would choose and thinks she still has the power to decide. More precisely, Soon et al. (2008) studied “free decisions” between many behavioral options using the multivariate pattern classification analysis (MVPA) which, combined with fMRI, allows one to identify specific contents of cognitive processes. “A pattern classifier, usually adopted from machine learning, can be trained on exemplars of neural patterns acquired when participants make different decisions and can learn to distinguish between these. If the activation patterns contain information about the decisions, the trained classifier can then successfully predict decision outcomes from independent data” (Bode et al., 2014).

In Soon et al.’s (2008) experiment, subjects carried out a freely paced motor-decision task (choosing to press a button with either the left or the right index finger) while their brain activity was being measured using fMRI. The subjects then had to report the moment of the decision, not by using a clock as in Libet’s experiment, but by selecting a letter in a stream that was being presented during the task. Soon et al. (2008) used fMRI signals to find local neural patterns and draw from such patterns all possible information decoded second by second thanks to the statistical techniques of pattern recognition. The brain areas that were mostly involved in the performance of the actions are the primary M2 and the SMA, while two other brain regions encoded the subject’s motor decision ahead of time and with high accuracy. Indeed, the frontopolar cortex (BA10) and a portion of the cingulate cortex can be monitored to understand what kind of choice will be made by the person before they are conscious of having taken a specific decision in the task they were given. The prediction can be made, with a relevant approximation (60% mean accuracy), up to 7 s before the conscious choice is experienced by the subject, thanks to the fMRI signals detected in the BA10 (one should take into account that the subjects are asked to think hard about the choice before making it, whereas usually simple choices do not require long subjective reflection). “The temporal ordering of information suggests a tentative causal model of information flow, where the earliest unconscious precursors of the motor decision originated in frontopolar cortex, from where they influenced the buildup of decision-related information in the precuneus and later in SMA, where it remained unconscious for up to 10 s” (Soon et al., 2008).

This seems to revive the old issue of God’s foreknowledge that forced theologians to wonder if man can be considered free, if someone already knows his future choices. Indeed, the authors speak of “free” decisions determined by brain activity ahead of time by placing “free” between inverted commas, as freedom is taken to be a commonsensical hypothesis. In this regard, the authors claim: “we found that the outcome of a decision can be encoded in brain activity of prefrontal and parietal cortex up to 10 s before it enters awareness. This delay presumably reflects the operation of a network of high-level control areas that begin to prepare an upcoming decision long before it enters awareness” (Soon et al., 2008).

Another interesting study is that conducted by Alexander et al. (2016): using a new experimental design, it found that the RP also occurs in the absence of movement. It suggests that “the RP measured here is unlikely to reflect preconscious motor planning or preparation of an ensuing movement, and instead may reflect decision-related or anticipatory processes that are non-motoric in nature” (Alexander et al., 2016). The experimental design used a modified version of Libet’s task. Subjects had to choose between four letters whenever they wanted, by taking note of the exact moment of their choice. Later, in half the trials, the subjects had to push a button as soon as they made the decision, whereas in the other half subjects had to do nothing to mark their choice. At the end of the task, all subjects had to report when they had made their decision. In this way, by EEG, electrooculography (EOG) and electromyography (EMG), it was possible to see the RP of the decision-making both in motor and non-motor contexts.

The authors did not find any strong differences between the two RPs, thereby affirming that there is a pure cognitive contribution to RP that does not reflect processes related to movement. They thus suggest that cognitive RP might reflect action preparation, general anticipation and spontaneous neural fluctuations. Interestingly, they exclude that the RP reflects action preparation since it is a non-motor processing. And as to anticipation they cannot exclude that RP may be specifically associated with free choice. So the RP could merely reflect the average of spontaneous fluctuations (see “Other Neuroscientific Hypotheses on Free Will” Section).

## Free Will as an Illusion

All these experiments seem to indicate that free will is an illusion. Yet, these relevant experiments can be interpreted in many ways. A possible view is that, in some way, determinism can be observed directly within ourselves. This interpretation might lead to the conclusion that free will is just an illusion. In fact, if one considers as a condition of free will the fact that it should be *causa* *sui* (i.e., it should be able to consciously start new causal chains), such a condition is incompatible with determinism as it is usually defined. For it, in fact, all events are linked by casual relations in the form of natural laws, which started long before we were born and which we cannot escape.

However, determinism has generally been regarded as a metaphysical claim, not refutable by empirical findings. One could properly talk of automatism in the brain, not of determinism, based on the evidence available. (In any case, endorsing indeterminism might lead to consider our behavior as the causal product of choices that every time produce different results, as if we rolled a dice. This doesn’t seem to make us any freer than if determinism were overturned; cf. Levy, 2011). Most importantly, another feature of freedom seems to be a pure illusion, namely the role of consciousness. The experiments considered thus far heavily question the claim that consciousness actually causes voluntary behavior. Neural activation starts the decisional process culminating in the movement, while consciousness “comes after”, when “things are done”. Therefore, consciousness cannot trigger our voluntary decisions. But the role of consciousness in voluntary choices is part of the definition of free will (but the very definition of consciousness is a matter of debate, cf. Chalmers, 1996).

Empirical research in psychology also shows that our mind works and makes choices without our conscious control. As proposed by psychologist Wegner (2002, 2003, 2004) and Aarts et al. (2004), we are “built” to have *the impression* to consciously control our actions or to have the power to freely choose, even though all that is only a cognitive illusion. Many priming experiments show that people act “mechanically” (even when their behavior might appear suited to the environment and even refined). Automatic cognitive processes, of which we aren’t always aware, originate our decisions, and they were only discovered thanks to the most advanced scientific research. Ultimately, consciousness, which should exercise control and assess the reasons for a choice, is thus allegedly causally ineffective: a mere epiphenomenon, to use the terminology of the philosophy of mind. This is what has been called *Zombie Challenge*, “based on an amazing wealth of findings in recent cognitive science that demonstrate the surprising ways in which our everyday behavior is controlled by automatic processes that unfold in the complete absence of consciousness” (Vierkant et al., 2013).

These experiments have triggered a huge debate and led scientists, philosophers and intellectuals to claim (or insist even more, if they already denied free will) that free will doesn’t exist (Greene and Cohen, 2004; Cashmore, 2010; Harris, 2012). It seemed as though neuroscience had produced empirical evidence against free will, so that the century-long debate on it could be considered solved. However, Libet’s experiments have been also criticized. Much criticism was directed to the philosophical interpretation of these studies (Mele, 2014) or to their theoretical assumptions (Nachev and Hacker, 2014), which are important but not relevant here. Among the forms of criticism, one has to mention the theories of action that separate the deciding from the initiating (Gollwitzer, 1999; Gollwitzer and Sheeran, 2006). In that case, free and conscious deliberating could still have a relevant casual role, long before the actual performance of the action.

Other objections, more markedly neuroscientific, were made for instance by Trevena and Miller (2010). They argued that the RP is not an intention to move, but only indicates that an attentional process is in place in the brain, since when subjects “attended to their intention rather than their movement, there was an enhancement of activity in the pre-SMA” (Lau et al., 2004). In any case, “there was no evidence of stronger electrophysiological signs before a decision to move than before a decision not to move, so these signs clearly are not specific to movement preparation”, (Trevena and Miller, 2010). Others have noted that the introspective estimates of event timing are disputable or inaccurate, and measures in general are not sufficiently exact (Dennett, 1984a,b, 2003).

## More than Explaining Away

Other studies using multivariate pattern analysis with EEG confirmed that the subjectively free decisions might be made in the brain in the same way as evidence-based perceptual decisions (Bode et al., 2012a,b, 2013). Indeed, Bode et al. (2012b) wrote,

we directly decoded choice-predictive information from neural activity before stimulus presentation on pure noise trials on which no discriminative information was present. Choice behavior on these trials was shown to be primed by the recent choice history. Modelling of sequential effects in RT and accuracy confirmed that such choice priming biased the starting point of a diffusion process toward a decision boundary, as conceptualized in evidence accumulation models of perceptual decision making (Bode et al., 2012b).

In other words, the authors found that internally (and maybe stochastically) generated neural activity can bias decisions that are expected to be stimulus-responsive or (possibly) reason-responsive. In this case, as in others that I will consider below, the understanding that we begin to have of the neuronal processes in play shows us that there is a complexity of factors at work. Some of these factors seem to be genuinely random, due to the pure noise produced by the default brain activity, while other factors can be traced back to the previous history of decisions taken in similar situations or related to the present one. Therefore, there is no “mysterious” start of the action as a linear process that, from the initial command, is then executed, as in Libet’s simplified model. Rather, this outcome is the result of a multiplicity of causal elements, which are homogeneous from the viewpoint of proximal mechanisms but of different relevance from the viewpoint of interpretation in terms of intentional psychology.

Another study has shown that attempts to account for (make sense of) insufficient perceptive clues use the same neural networks as those involved in “free” decision-making (Bode et al., 2013). An fMRI-based pattern classifier can be trained to differentiate between different perceptual guesses and try to predict the outcome of non-perceptual decisions, like those made by the participants in the experiments considered so far. Specific activation patterns detected in the medial posterior parietal cortex have allowed the authors to make correct predictions on the participants’ free choices based on the previously decoded perceptual guesses decoded, and the other way round.

The task was the following: the participants were given a masked stimulus and had to say what category the stimulus belonged to. They had to freely choose among many categories. Thanks to the multivariate pattern analysis it was possible to identify the model of “free decisions” to make correct predictions in the context of perceptual judgments and identify the model of the “guess decisions”, to make correct predictions in the context of “free decisions”. It thus seems that a similar neural code for both types of decision is present. In those cases one could say that guessing is similar to making a free decision, since the brain, in the absence of sufficient external cues, has to decide internally. So perceptual decisions can be predicted from specific preceding neural activity when the brain doesn’t have enough internal elements to reach the threshold of perceptive decision.

Studies and commentaries have nevertheless drawn attention to possible confounds and bias in those experiments, namely they might be affected by previous choices with a form of auto-correlation in spontaneous decisions. In particular, Lages and Jaworska (2012) “trained a linear classifier to predict “spontaneous decisions” and “hidden intentions” from responses in preceding trials and achieved comparable prediction accuracies as reported for multivariate pattern classification based on voxel activities in frontopolar cortex”. Lages et al. (2013) have stressed a possible sequential information processing between trials that can introduce a confound, and recommended that “rather postulating a 50% chance level, prediction should be tested with a permutation test and/or separate multivariate classification analyses conditional on the previous response”.

The prediction of perceptual decisions from specific preceding neural activity is linked to what is defined “evidence accumulator model for free choice” (Bode et al., 2014). The explanation starts with the fact that predictive activation patterns preceding decisions become increasingly similar to the patterns detected when the decision is consciously experienced by the subject. This could mean that a slow build-up of decision-related activity occurs, as it happens in accumulation of decision-related evidence to a decision threshold (Ratcliff, 1978; Ratcliff and McKoon, 2008). Also, as already noted, when no external feedback is available, the previous choice is used as external feedback (Akaishi et al., 2014). The history of previous decisions has a systematic effect on subsequent choices, related to the activity in medial posterior parietal cortex/posterior and posterior cingulate cortex (Bode et al., 2011, 2013). And the systematic effect can go in the direction of repetition or of avoidance of repetition depending on the task (Mochizuki and Funahashi, 2014).

Here is an important point that deserves study from the neuroscientific point of view but also from that of a philosophical interpretation of free will. It consists in the fact that the internally generated brain activity has to do both with the stochastic noise and with the history of the subject’s choices. On the one hand, the stochastic noise comes both from the configuration that the brain has on average as a result of evolution (adaptive significance) and from individual development, resulting from random processes and environmental influences. On the other hand, the history of the choices is derived from the same process (in part stochastic) that I have just described.

In any case, if (at least some) very short-term decisions have a genesis similar to that described here, these decisions contribute to shaping the brain activity, and then, presumably, also to influencing decisions on a longer time scale that it is not yet possible to investigate experimentally. Ultimately, this could mean that there is a confluence of causal factors at the level of microdecisions. These factors add up in a way that it is hardly possible to tackle for current science. Then also the reasons motivating an action, typical of free actions, such as “I punched the stalker because it is right to punish those who behave in this way and because I wanted to set an example for all”, encoded in neural activity, can be part of the sum of neural causes.

In fact, experimental psychology has been trying to take into account long-term influences. In the so-called marshmallow experiment, researchers focused on delayed gratification (Mischel et al., 1972, 1989). A child was given a choice between one small, immediate reward and two small rewards (i. e. a larger reward) if they were able to wait some minutes while the psychologist left the room and then came back. Children who waited longer for the their rewards tended to have better life outcomes and accomplishments. Such experiments are relevant in terms of explanations and predictions, but it seems hard to trace behavioral profiles back to specific profiles of cerebral activation, once we are aware of the complexity of causal chains in the evidence accumulation model.

As Bode et al. (2014) write, in the hypothesis of an evidence accumulator that collects sensory evidence until a decision threshold is reached,

task instructions, participants’ internal motivation, and previous choices all have a strong influence on how decision tasks are performed when external information is either unavailable (as in free decisions), or unhelpful (as in perceptual guessing). In the case of free decision tasks, fluctuating intention for one or the other option may result from active competition between neural representations of both options in decision networks (or rather although not consciously monitored by the participants, the previous choice history, embodied in dynamic states of decision networks, can become the primary determinant of behavior, simply because nothing else is available (Bode et al., 2014).

However, in this way things get more complicated and at a macroscopic level of behavioral observation, this blurs but doesn’t do away with the idea of free behaviors and behaviors that could be taken as unconscious decisions, of which we become aware only when the action has been performed. What remains to be solved is the problem of the distinction between external stimuli that trigger a stimulus-response circuit, and internal self-paced intentions and decisions that trigger voluntary circuit (Haggard, 2008).

## Other Neuroscientific Hypotheses on Free Will

### Beyond Determinism and Consciousness

The concept of free will relevant to our moral and legal, personal and social practices is much more complex than that captured by the experiments considered up to now. But here what matters are not so much theoretical considerations or those derived from experimental psychology (such as the role played in decisions by implementation intentions, which then re-evaluate the active role of consciousness; Gollwitzer, 1999), but those that originate from the neuroscientific research itself. In what might be called a new phase of empirical investigation on free will, the problem of determinism and the role of consciousness is left in the background, and the focus goes to other factors that enter the brain mechanisms of decision-making, without asking first if those processes (necessarily the most simple, at least for now) are deterministic or stochastic. On the other side, neuroscientists are trying to confine the concept of free will to operationalizable situations, so as to measure it and be able to identify, at least as a goal, its neural correlates.

There is a line of research on non-human primates, but more recently also on humans, which studies fine decision-making at the neuronal level, bringing it back to a mechanistic process that might be the neuronal interface of our common sense descriptions. This trend has been well described by Roskies (2010a,b, 2013), who is one of the major supporters of this approach. For example, in Shadlen and Newsome’s (2001) experiments, monkeys are trained to look at stimuli consisting of points that move randomly to the right and to the left and to “indicate” the overall direction of the points. The monkeys give this indication moving their eyes (with a saccade) to the right or to the left. What emerges is that the activity of the neurons of the lateral interparietal (LIP) area increases with the information in the sensory cells of the middle temporal (MT) area and upper middle temporal (MST) area. The discharge rates rise up to reaching a given level, at which the monkey performs the saccade and the neurons stop discharging. This is the threshold for a decision to take place. The time taken to reach the threshold level depends on the perceptual characteristics of the stimulus (the strength of the movement over time) and the discharges stop after the answer was given.

The discharges also depend on whether the monkey is asked to answer when he wishes, or rather to hold back the response until the signal is given for the saccade. If the monkey is asked to wait until the signal is given to respond, LIP neurons continue to discharge even in the absence of the visual stimulus (Gold and Shadlen, 2007). According to Roskies (2010b), this is the discharge scheme of a neuron involved in the decision-making process; the levels of discharge can be maintained in the absence of the stimulus, signifying the independence of the decision from the inputs on which it operates, and the activity continues until it reaches the critical level at which the response is generated, or until the neurons that represent the elements accumulated in favor of a different choice lead to eye movement. In addition, electrical stimulation of LIP neurons can influence the monkey’s decision, indicating that LIP cells causally contribute to the process that triggers decision and action (Hanks et al., 2006). It remains, however, to be established whether this role is that of deliberation that leads to a decision or that of the decision itself.

The reaction times and the accuracy in the evaluation are very similar between monkeys and humans, with the probability of choice and the response time connected in a similar way to the difficulty of discriminating the stimulus, so that it can be assumed that also in humans these neural processes are similar. A mathematical description of the dynamics of this system allows one to talk about the race towards the critical threshold (Gold and Shadlen, 2007; Wong et al., 2007). According to this model, the neuronal populations with specific response properties represent different “hypotheses”. The discharge rates represent the strength of the evidence in favor of those hypotheses based on evidence gathered from the environment. When the evidence for and against each hypothesis is integrated, the discharge rates reach or move away from the critical level, which represents the decision point. This is the point at which the animal “made a choice” about the overall direction of movement. The first group that reaches this threshold “wins”, leading the motor response.

Schurger et al. (2012) proposed a different interpretation of the premovement buildup of neuronal activity preceding voluntary self-initiated movements in humans as well. They used “a leaky stochastic accumulator to model the neural decision of “when” to move in a task where there is no specific temporal cue, but only a general imperative to produce a movement after an unspecified delay on the order of several seconds”. According to their model, “when the imperative to produce a movement is weak, the precise moment at which the decision threshold is crossed leading to movement is largely determined by spontaneous subthreshold fluctuations in neuronal activity. Time locking to movement onset ensures that these fluctuations appear in the average as a gradual exponential-looking increase in neuronal activity” (Schurger et al., 2012).

The model proposed by Schurger et al. (2012) accounts for the behavioral and EEG data recorded from human subjects performing the task and also makes a specific prediction that was confirmed in a second electroencephalography experiment: fast responses to temporally unpredictable interruptions should be preceded by a slow negative-going voltage deflection beginning well before the interruption itself, even when the subject was not preparing to move at that particular moment. The task was to repeatedly push a button, sometimes at will, sometimes in response to a sound produced by the experimenters according to a causal sequence. The speed of response (pressing the button) when the sound is produced is related to the proximity to the peak of the background brain activity, which appears to be random, an ebb and flow that has its highest point in the threshold at which it produces the decision to push the button.

According to this explanation, “the RP does not reflect processing within a specific action domain. Our finding that movement does not significantly modulate RP amplitude supports this aspect of their claim by extending the RP to the domain of covert decisions” (Alexander et al., 2016). Another consequence is the fact that the neural decision to move at a specific time happens much later compared to Libet’s hypothesis, and the RP is only a by-product of a drift diffusion process. But the RP would still be predictive in that it precedes action and conscious awareness of both motor and cognitive action. However, the RP is predictive with regards the whether and the when, if a known task is performed, but not with regards to the what of the action (Brass and Haggard, 2008).

Jo et al. (2013) seems to go in the same direction with their work: they considered both the positive and the negative potential shifts in a “self-initiated movement condition” as well as in a no-movement condition. The comparison of the potential shifts in different conditions showed that the onset of the RP appeared to be unchanged. “This reveals that the apparently negative RP emerges through an unequal ratio of negative and positive potential shifts. These results suggest that ongoing negative shifts of the SCPs facilitate self-initiated movement but are not related to processes underlying preparation or decision to act” (Jo et al., 2013).

Murakami et al. (2014) confirmed those findings. They used rats, who had to perform a specific task: wait for a tone (which was purposely delayed) and decide when to stop waiting for it. The rats’ neuronal activity of the secondary M2 was recorded and resulted consistent with the model of integration-to-bound decision. “A first population of M2 neurons ramped to a constant threshold at rates proportional to waiting time, strongly resembling integrator output. A second population, which they propose provide input to the integrator, fired in sequences and showed trial-to-trial rate fluctuations correlated with waiting times” (Murakami et al., 2014). Also, an integration model based on the recorded neuronal activity in the considered brain areas has allowed the researchers to quantitatively foresee the inter-neuronal correlations manifested during the task performance. “Together, these results reinforce the generality of the integration-to-bound model of decision-making. These models identify the initial intention to act as the moment of threshold crossing while explaining how antecedent subthreshold neural activity can influence an action without implying a decision” (Murakami et al., 2014).

Schurger et al. (2016) stress that the main new finding about the brain activity preceding SVM “is that the apparent build-up of this activity, up until about 200 ms pre-movement, may reflect the ebb and flow of background neuronal noise, rather than the outcome of a specific neural event corresponding to a “decision” to initiate movement”. The model used is the bounded-integration process, “a computational model of decision making wherein sensory evidence and internal noise (both in the form of neural activity) are integrated over time by one or more decision neurons until a fixed threshold-level firing rate us reached, at which the animal issues a motor response. In the case of spontaneous self-initiated movement there is no sensory evidence, so the process is dominated by internal noise” (Schurger et al., 2016). The stochastic decision model (SDM) used by Schurger et al. (2012) allowed them to claim that bounded integration seems to explain stimulus-response decision as relying on the same neural decision mechanism used for perceptual decisions and internal self-paced intention and decision as “dominated by ongoing stochastic fluctuations in neural activity that influence the precise moment at which the decision threshold is reached” (Schurger et al., 2016). And this mechanism seems to be shared with all animals including crayfish (Kagaya and Takahata, 2010).

The philosophical implications could be that “when one forms an intention to act, one is significantly disposed to act but not yet fully committed. The commitment comes when one finally decides to act. The SDM reveals a remarkably similar picture on the neuronal level, with the decision to act being a threshold crossing neural event that is preceded by a neural tendency toward this event” (Schurger et al., 2016).

### The Veto Power

Another recent study has brought back to the center of neuroscientific research the space of autonomy that the subject seems to have compared to the idea of free will as an illusion supported by the experiments based on the alleged unconscious onset of the action. Schultze-Kraft et al. (2016) showed that people are able to cancel movements after elicitation of RP if stop signals occur earlier than 200 ms before movement onset. In the real-time experiment, “subjects played a game where they tried to press a button to earn points in a challenge with a brain–computer interface (BCI) that had been trained to detect their RPs in real time and to emit stop signals” (Schultze-Kraft et al., 2016).

The subjects had to press with their foot a button on the floor after a green light flashed: they could so whenever they wanted after about 2 s. Participants earned points if they pressed the button before the red light to come back (the stop signal). The experiment was composed of three phases. In the first phase, the stop signals were lit at random and the movements of the subjects were not predicted. In the second phase, the authors used data taken from the EEG on the participants in the first phase. In this way a classifier was trained to predict (with imperfect accuracy) the movements (the When and the Whether, not the What). In this phase, the BCI could foresee the fact that the subject would press the button thanks to the detection of the RP and therefore turned on the red light to earn points against the subject if it could not stop the movement. In the third phase, the subjects were informed that the BCI could “see their preparation of the movement” and they had to try to beat the computer by moving in an unforeseeable way.

In all phases of the experiment, there was no difference between RPs. While in the first phase, in 66.5% of the cases, subjects were winning by pressing the button with the green light on, in stages two and three trials in which subjects were able to beat the computer, by not pushing the button with the red light on, decreased to 31%, and warning participants of the prediction of the BCI would not help them do any better. The authors could thus claim that “despite the stereotypical shape of the RP and its early onset at around 1000 ms before EMG activity, several aspects of our data suggest that subjects were able to cancel an upcoming movement until a point of no return was reached around 200 ms before movement onset. If the stop signal occurs later than 200 ms before EMG onset, the subject cannot avoid moving” (Schultze-Kraft et al., 2016). The explanation of the minimum threshold of 200 ms could reflect the time necessary for the stop signal to light up, the subject to perceive it and cancel the movement that was already being prepared.

As to which cortical areas are involved in vetoing an already initiated movement, some studies have tried to identify them. Brass and Haggard (2007) examined the voluntary inhibition using an experimental paradigm that was based on the Libet task. The subjects were asked to press a button while watching a cursor moving along the face of a clock. Every time, after pressing the button, the subjects had to signal the precise moment when they thought they decided to press the button. In addition, the instructions specified that the participants had to inhibit the execution of the response in some tests of their choice. Comparing this voluntary inhibition condition with the condition in which the action had not been inhibited, the authors observed an activation of the dorsal fronto-medial cortex (DFM). This area is different from the brain regions involved in the stop signal tasks, in which the inhibition is controlled by external signals. Furthermore, the DFM cortex is also distinct from the brain regions controlling the activity linked to the when and what components of voluntary action. Brass and Haggard (2007) have interpreted this finding as evidence that there is a mechanism of voluntary inhibition that can be dissociated, in neuroanatomo-functional terms, from an “environmental” inhibiting mechanism, which involves the lateral prefrontal cortex.

This finding was replicated in a subsequent study of Kühn et al. (2009), in which the subjects had to avoid dropping a ball sliding down a ramp, by pressing a button before the ball came down and broke. In some tests of their choice, they could choose to voluntarily inhibit the response. The comparison of the condition of voluntary inhibition with the condition of voluntary action still showed activation of the DFM cortex, supporting the idea that this area is involved in the inhibition of voluntary action (Schel et al., 2014).

Finally, Schultze-Kraft et al. (2016) declared to be agnostic about the interpretation of their data in regards of RP. As the RP is predictive of the subsequent movement, it could be read as “the leaky integration of spontaneous fluctuations in autocorrelated neural signals”. Theoretically, the question remains about the departure of the intention to block the action while the movement is being prepared, along with the possible coexistence of two intentions suggested by the commands of the experimenters. The participants in the experiment, in fact, want to win against the computer, therefore they want to push the button, and also have the intention, partly contrasting, not to push the button when the computer turns the red light on.

### A More Realistic Model

This novel perspective offered by the line of research by Schurger et al. (2012) here described works on very simple decision-making processes and could be exposed to the same criticism in this regard have been made to Libet’s research line. But Roskies (2010b) has suggested some tracks along which to develop research on more complex decision-making processes, close to those relevant to social life. First, one must introduce the value of the decision, seen as a subjective or moral feature that drives action. By manipulating the expected rewards for correct action or for a particular type of decisions, or by manipulating the probabilities of the outcomes, both the decision and the activity levels of LIP neurons are altered (Platt and Glimcher, 1999; Glimcher, 2002; Dorris and Glimcher, 2004; Sugrue et al., 2004). In this way it is possible to change the monkey’s choice about the objective of the saccade by offering her favorite reward. Although it is not known how the figures are represented, it seems that the Lip neurons can integrate the information on the value or on the reward in the decision-making process, and that information has a causal role.

As for the reasons, and the responsiveness to them, Roskies (2010b) suggests that also the reasons, albeit discursive and propositional, may be encoded as information at the neuronal level. Simplifying, in her view one might think that in a situation where, say, there is little food and many people, different populations of neurons represent the content “I am hungry”, while others represent “others need this more than I do”, others “the weak come first” and so forth, weighing reasons in terms of activation and modulation of the response of the populations of neurons delegated to the choice and the final decision. However, such a model (Dorris and Glimcher, 2004) should be considered as purely hypothetical because first we do not know what are the specific populations of neurons, we don’t yet have the instruments to identify them, and we do not know their interactions (also considering the recent failures of naturalized semantics).

Secondly—and perhaps most importantly—it is unclear how what we externally call “reasons” could be activated and weighed by the decision-maker understood as a unitary subject or self, according to the description for which we truly act based on reasons. In this case, I believe one cannot seek a simple neural interface for commonsensical concepts and notions. In fact, the idea of a deep and unitary self—the idea of a conscious subject controlling her behavior instant by instant—has been strongly challenged by evidence coming from empirical psychology and cognitive neuroscience (Dennett, 1991; Metzinger, 2004, 2009). Therefore, one should avoid the temptation to reproduce such a description in neural levels. But if we trace back the reasons to populations of neurons in a mechanistic model—if we trace them back to thresholds—it is not easy to figure out who makes the decisions and why. If it is true that some people seem to be more sensitive to specific reasons, other than those to which other people are sensitive, and if people can change over time the reasons by which they are usually motivated, and in certain situations the same people may not to respond to the reasons to which they are usually sensitive, one has to wonder if what prevails are processes that we would call random or that, in any case, are beyond our control.

Here the role of consciousness seems again to be relevant. If experiments à la Libet seemed to have ruled it out from a causal standpoint, the experiment by Schultze-Kraft et al. (2016) on movement vetoing seems to reassess its role in blocking the preparation process triggered in the brain. In this sense, this seems to be a more realistic line of neuroscientific investigation on free will, one that contemplates, even in broad terms, stochastic brain processes, for the most part triggered by environmental stimuli, which often are not aware of (the same as our train of thoughts arising spontaneously without us being able to orientate it from the beginning), but also by spontaneous activity of the brain (Changeux, 2004; Brembs, 2011) that creates models of reality. “Learning mechanisms evolved to permit flexible behavior as a modification of reflexive behavioral strategies. In order to do so, not one, but multiple representations and action patterns should be generated by the brain” (De Ridder et al., 2013). And this repertoire is not infinite. Indeed, “our evolutionary-evolved brain potential to generate multiple action plans is constrained by what is stored in memory and by what is present in the environment” (De Ridder et al., 2013). Schurger and Uithol (2015) also argue that the “actions emerge from a causal web in the brain” and that the “proprioceptive feedback might play a counterintuitive role in the decision process”. They, thus recommend the use of dynamical systems approach for the study of the origins of voluntary action.

On these spontaneous processes we can exercise control, which can be considered automatic and unconscious when evaluated with the classical theoretical criteria of conscious control. First, there is an innate behavioral repertoire of provisions linked to survival in the environments within which we evolved. Secondly, there is a repertoire of behavioral provisions that is stratified in terms of conscious repetitions due to environmental stimuli or to internal choices (with all the limitations that this expression has in reference to the brain mechanisms analyzed so far) and then becomes automatic. The control can, however, also be explicit, with obvious limitations and cases of complete control failure. Based on this complex self-construction (which has a neural correlates), we are creatures with a higher or lower degree of free will. This free will may then be better understood and circumscribed, so as to be more objectively operationalized and also measured.

## Operazionalizing, Measuring and Verifying: From the Action to the Brain

My view is that a richer conceptualization of free will—one that is able to overcome the stall of the metaphysical debate as well as the current difficulties of neuroscience (Nachev and Hacker, 2014) and empirical psychology (Nahmias, 2014)–has to be linked to the idea of “capacity”. In fact, as claimed by Mecacci and Haselager (2015), the kind of free will investigated by neuroscientific experiments, which is self-generated and defined according to the absence of cues, “does little justice to the common sense practice of holding people responsible for their freely willed actions that consists in asking explanations and justifications from the actor” (Mecacci and Haselager, 2015).

Another important point is that there are differences in time scales between laboratory tasks (the milliseconds to seconds time range) and real life or, better, life as we measure it temporally (seconds, minutes, hours, weeks, years) regarding decisions that really concern us. Even if the underlying mechanism might be the same, the experiments described so far cannot investigate whether decisions with a longer maturation process are free and to what extent they are such. It might be possible to distinguish between proximal and distal mechanisms, but this doesn’t seem feasible lacking the tools to address decisions involving longer time scales. For this reason it might be useful to introduce other and different ways to conceptualize and operationalize (supposedly) free actions.

“By capacity, in the context of free will, we mean the availability of a repertoire of general skills that can be manifested and used without the moment by moment conscious control that is required by the second condition of free will we have previously seen” (Lavazza and Inglese, 2015). The concept of capacity is related to that of internal control, understood as the agent’s “ownership” of the mechanism that triggers the relevant behavior and the reasons-responsiveness of that mechanism (Fischer and Ravizza, 2000). And reasons-responsiveness must involve a coherent pattern of reasons-recognition. “More specifically, it must involve a pattern of actual and hypothetical recognition of reasons that is understandable by some appropriate external observer. And the pattern must be at least minimally grounded in reality” (Lavazza and Inglese, 2015). The concept of capacity used in this sense, and combined with the idea of reasons-responsiveness, also avoids the objection of determinism that has always weighed on the debate on free will. From a philosophical point of view, the approach related to capacity may fall indeed in the strand of so-called compatibilism, which defends the fact that human freedom can exist even if determinism is true of the physical world.

Cognitive abilities might be firstly operationalized as a set of neuropsychological tests, which can be used to operationalize and measure specific executive functions, as they are strongly linked to the concept of control. Executive functions, also known as control functions, are essential to organize and plan everyday behavior—which is not the instant behavior found in Libet’s experiments. Those skills are necessary to perform the greater part of our goal-oriented actions. They allow us to modulate our behavior, control its development and change it according to the environmental stimuli (the environment being both physical and social). Also, executive functions allow us to change our behavior based on it effects, with a sophisticated feedback mechanism; finally, they are also necessary for tasks of abstraction, inventiveness and judgment. Those who, for whatever reason, have a deficit in their executive functions cannot respond to their social environment appropriately, and struggle to plan their behavior or to choose between alternatives based on their judgment or interest. Sufferers of these deficits in executive functions often fail to control their instinctive responses and to modify their regular courses of action, or are unable to concentrate or persist in the pursuit of a goal (Barkley, 2012; Goldstein and Naglieri, 2014).

In general terms, the executive functions refer to the set of mental processes necessary for the development of cognitive-behavioral patterns adaptive in response to new and demanding environmental conditions. The domain of executive functions includes (Lavazza and Inglese, 2015):

- the ability of planning and evaluation of effective strategies in relation to a specific purpose related to the skills of problem-solving and cognitive flexibility.
- inhibitory control and decision-making processes that support the selection of functional response and the modification of the response (behavior) in relation to changing environmental contingencies.
- attentional control referred to the ability to inhibit interfering stimuli and to activate the relevant information.
- working memory referring to the cognitive mechanisms that can maintain online and manipulate information necessary to perform complex cognitive tasks.
- (and it can be added with regards to free will) creativity and the ability to cope with environmental changes through novel solutions.

Those of empirical psychology are higher order concepts, which act as a bridge between free will, which is something that is not in the brain but can be observed in behavior (along with its causes), and the underlying brain processes. It has been convincingly suggested that in the construction of a hierarchy of mechanisms and explanations (Craver, 2007), also to guide the exploration, one must go from inside to outside and from outside to inside. One goes from measurable skills to their brain basis, and from the tentative index of free will to the underlying (real) mechanisms.

Based on the evidence presented, I believe that a viable proposal is to construct an index related to compatible tests whose relevance can be uniformly ascertained. It would be a kind of IQ-like profile that would allow for the operationalization and quantification of a person’s cognitive skills. All the tests used (for example, Stroop Test, Wisconsin Card Sorting Test, Weigl’s Color-Form Sorting Test, Go-No Go Test) should be related to the subject’s age and education and then transformed in new standardized scores (Equivalent Scores, ES) on an ordinal scale, e. g. ranging from 0 to 4, with 0 representing scores below cut-off point and 4 representing scores equal to or better than average. Specific standardized scores exist in many countries or linguistic areas. The subjects would get for each test a raw score (or RS), given by the sum of the scores obtained in each item that makes up the test, which would then be standardized.

A synthetic index such as the one here proposed measures a certain range of cognitive and behavioral control skills that configure a certain kind of free will at the psychological-functional level. These are potential capacities measured with standardized instruments in laboratory situations, which do not consider any other factors that may restrict the freedom of a subject in specific situations, such as those that are relevant in moral scenarios and legal contexts. The same goes for moral judgment. However, an index such as the one I’m proposing here could be the first step, albeit certainly imperfect, towards more objective measures to discriminate between people who have more or less “free will” or, in other words, are more or less capable of self-control and rational choice (i.e., a reasons-responsive choice).

This hypothesis would be in line with the proposals of operationalizing free will advanced so far. According to Vohs (2010), freedom might be conceived of as the sum of executive functions and goal-directed, future-oriented behaviors, which include rational choice, planning, intelligent thought, and self-control. Free will can be then constituted by a limited stock of energy, devoted to guiding executive functioning processes. The free will index I am proposing is also consistent with Baumeister’s contribution:

Psychologists should focus on what we do best: collecting evidence about measurable variance in behaviors and inner processes and identifying consistent patterns in them. With free will, it seems most productive for psychologists to start with the well-documented observation that some acts are freer than others. As already noted, dissonance, reactance, coping with stress, and other behaviors have been shown in the laboratory to depend on variations in freedom and choice. Hence, it is only necessary to assume that there are genuine phenomena behind those subjective and objective differences in freedom. In a nutshell, we should explain what happens differently between free and unfree actions (Baumeister, 2008).

Empirical research on how human beings work has recently focused on self-control as a feature of free will. Self-control can be defined as the exertion of willpower on behavior. Self-control is thus generally regarded as the capacity to override inappropriate impulses and automatic or habitual responses and to suppress or delay immediate gratification so as to reach a long-term goal (Gailliot and Baumeister, 2007). “Being in control” includes the capacity to maintain goals, to balance long- and short-term values, to consider and evaluate the consequences of a planned action, and to resist being “carried away by emotion” (Churchland, 2006). Self-control can also be regarded as the ability of higher-order functions to modulate the activity of lower-level functions, where higher-order functions manifest themselves externally in complex behavior, adjusted according to the environmental needs, while lower-level functions are manifested in simple and stereotyped behaviors, not adjusted according to the demands of the environment (Roskies, 2010a). Everyone exhibits a different degree of self-control compared to other individuals, and for each person the degree of self-control varies over time (Baumeister et al., 2006; Casey et al., 2011; Dang et al., 2015). The variability of self-control that is manifested in behavior and can be measured with the test has its base in neuronal functioning, which in turn depends on education and habits, external circumstances and the internal neuronal noise.

However, two executive functions turn out to be central:

(i) the ability to predict the future outcomes of a given action; and (ii) the ability to suppress inappropriate, i.e., not sufficiently valuable, actions. Importantly, these two executive functions operate not only during the genesis of an action, but also during the planning of an already selected action. In fact, during the temporal gap between the time when an action has been chosen and the moment when the motor output is going to be generated, the context might have changed, altering the computed value of the action and thus requiring a radical change of the planned motor strategy (Mirabella, 2014).

It seems that the peculiarity of our freedom at the cognitive level is the ability to modulate or block courses of action that environmental stimuli automatically or unconsciously arouse in us—a reproposal in different form of Libet’s *free won’t and* Schultze-Kraft’s *vetoing*. These psychological-functional indicators must then lead to their cerebral bases. For instance, one can consider a situation in which one’s needs are satisfied (or not) and the consequent motivation to act based on the evaluation process of the need satisfaction.

This is an essential process and one that is continuously performed by our motor system. In fact, in most places where we live, if not all, we are surrounded by tools whose sight automatically activates motor schemas that would normally be employed to interact with those objects. These actions are prompted by the features of the objects, the so-called *affordances* (Gibson, 1979). It has been shown that even the simple observation of pictures depicting affordable objects (such as graspable objects) activates a sub-region of the medial frontal cortex, the SMA, even when there is no requirement to actually act on those stimuli (Grèzes and Decety, 2002). These stimulus-driven activations are rapid, involuntary, and unconscious (Mirabella, 2014).

Environmental stimuli, in this case, can induce a subject to make specific choices through a priming process that exploits our action tendencies. Typically, individuals are able to control their behavior, but in some cases they fail to do so; for example if suffering from microlesions of the SMA, people have a tendency to invariably implement a certain type of action, even if the environment, both physical and social, does not require it (Sumner et al., 2007). In fact, “the suppression of a triggered action might be seen not as an active process, but rather as an automatic consequence of the evaluative procedure” (Mirabella, 2014). One could then say that those who have the ability to better monitor, control and direct their own behavior are “freerer” than those who do not have this capability. Individuals affected by disorders of the executive functions are not able to grasp and process environmental stimuli to direct their behavior. For example, these people may not be able to stop the utilization behavior, an automatic mechanism that tends to make us interact with all the objects that are in our perceptual sphere.

Churchland (2006) and Suhler and Churchland (2009) proposed a hypothesis concerning the neural basis for control, which can bridge the gap between higher-order concepts and brain mechanisms. As she wrote,

Perhaps we can identify various parameters of the normal profile of being in control, which would include specific connectivity patterns between amygdala, orbitofrontal cortex, and insula, between anterior cingulate gyrus and prefrontal cortex, and so forth. Other parameters would identify, for each of the six non specific systems [identified via the neurotransmitter secreted at the axon terminals: serotonin, dopamine, norepinephrine, epinephrine, histamine and acetylcholine], the normal distribution of axon terminals and the normal patterns of neurotransmitter release, uptake, and co-localization with other neurotransmitters such as glutamate. Levels of various hormones would specify another set of parameters. Yet other parameters contrast the immature with adult pattern of synaptic density and axon myelinization. At the current stage of neuroscience, we can identify the normal range for these parameters only roughly, not precisely (Churchland, 2006).

This hypothesis would allow for specific brain correlates of a free will index based on the executive functions-guided self-control and even, hypothetically, a direct brain measure of being in control For example, a recent study (Bartelle et al., 2016) highlights the possibility of having MRI imaging of dopamine release thanks to a engineered protein that binds to the neurotransmitter and works as a MRI-visible probe. As the authors put it, “one could imagine a future in which molecular fMRI is used to determine brain-wide neurochemicals maps corresponding to a universe of stimuli and behavioral programs”. Even though one should always consider that there isn’t perfect correspondence between higher-order concepts and putative neural correlates.

In particular, one must consider that what matters in interpersonal relations and in law, to give two examples of practical relevance of free will, is freedom as actually performed: that is, freedom as it can be observed and with some approximation, also measured through a series of psychological tests. This does not mean that the same level of freedom manifested in behavior matches the same level of activation of the related brain areas. However, one can investigate the brain causes of “freedom deficit” compared with the average shown by relevant samples of the population, and so come to a progressive refinement of the research on the neural bases of free will.

Another example is given by the investigation of the role of the cholinergic interneurons in behavioral flexibility (Aoki et al., 2015). This class of neurons seem to be connected to survive in an ever-changing world, which requires behaving flexibly. Flexibility can be assessed (and measured) at a behavioral level, but cerebral mechanisms remain largely unknown. Using conventional tests on behavioral flexibility, which require animals to shift their attention from one stimulus property (e.g., color) to another (e.g., shape), researchers probed the effects of an immunotoxin-induced lesion of cholinergic interneurons in the striatum.

A selective cholinergic ablation was made by means of injections of immunotoxin, which targeted neurons containing choline acetyltransferase in the dorsomedial or ventral striatum. A control group was instead injected with saline. “When encountering a change of behavioral rules after the set-shift, either lesion made animals stick to a previously correct but now invalid response strategy. They also showed less exploratory behavior toward finding a new rule. Most interestingly, ablation of cholinergic neurons in the dorsomedial striatum impaired a shift of set when it required attention to a previously irrelevant cue. On the other hand, ventral cholinergic lesions had an effect on a shift in which a novel stimulus was introduced as a new directional cue” (Aoki et al., 2015). Animals thus can be taken to be “less free” when striatal cholinergic interneurons don’t work properly.

This last example serves to indicate how to bridge the gap between overt behaviors (to which we tend to attribute the property of freedom) with neuronal mechanisms that are clearly identifiable and even manipulable. In fact, it is not so important to look at the conscious aspect of a single proximal mechanism, but rather to consider the manifest behavioral effect that the considered mechanism helps to produce. This way there would be a paradigm shift with respect to the neuroscience research on free will, which seems to have long been too closely linked to the falsification of the theoretical assumption that an action is free only if it has a beginning that is fully controlled by a conscious process. The proposal, I am making here has only the ambition to be a potentially helpful contribution to theoretical debate and empirical research, although its limits are very clear. First, it focuses on a specific part of what is intuitively called “free will”, relating it to the idea of “capacity”. Second, it proposes to measure free will at a psychological level by means of a unitary index that inevitably misses many nuances of the notion and the relative capacity. Furthermore, the search for the neural correlated of such capacities implies not only the identification of causal mechanisms, but also the consideration of many cerebral areas. All of this makes things harder compared to approaches à la Libet. Nevertheless, there is manifest advantage: there is a greater degree of realism and adherence to the actual behavioral manifestation of what we call “free will”.

## Conclusion

Free will is an elusive but crucial concept. For many years we have known that the functioning of our brain has to do not only with the belief that we have free will but also with the existence of free will itself. Evidence of the unconscious start of movement, highlighted by the RP signal, has led to believe that we had reached an experimental proof of the non-existence of free will—which many already claimed at a theoretical level based on the argument of the incompatibility between determinism and freedom. Along with other evidence provided by experimental psychology, the branch of studies inaugurated by Libet has contributed to seeing free will as an illusion: this view seemed to be reliably supported by science, and in particular by neuroscience. Recent studies, however, seem to question this paradigm, which sees the initiation and conscious control of the action as the first requirement of free will, allegedly proving that there are no such things.

The stochastic models and the models of evidence accumulation consider decision as the crossing of a threshold of activity in specific brain regions. They do not restore the idea of conscious control but turn away from the previous paradigm. These studies cannot yet fully explain how the intention to perform an action arises in the brain, but they better account for the complexity of the process. In particular, they recognize the role of the spontaneous activity of the brain, of external cues and other factors—including those that might be called “will” and “reasons” (which, however, do not currently have precisely identified neural correlates)—in reaching the critical threshold. Studies that show how we can consciously block movements whose preparation has already begun unconsciously, then, indicate how the subject is able to exercise a form of control, whose genesis however is still unclear.

One could state that free “decision-making draws upon a rich history of accumulated information, manifested in preferences, attitudes and motivations, and is related to the current internal and external environment in which we act. Complete absence of context is impossible” (Bode et al., 2014). In this framework, I have here proposed to integrate neuroscientific research on free will by connecting higher-level concepts with their neural correlates through a psychological operationalization in terms of skills and cognitive functions that do not necessarily imply a continuous conscious control over the decision-making and action process. This may also allow one to create a quantitative index, albeit still quite rudimentary, of the degree of freedom of each subject. This freedom would be specifically defined and therefore may not perfectly coincide with the intuitive concept of free will. Starting from these functional indicators, which psychology has well clarified, one could then move on to investigate the precise neural correlates for a different and (possibly) more fundamental level of explanation in terms of brain processes that enable the executive functions.

According to Craver (2007), a mechanistic explanation is able to lead to an inter-field integration. There are two relevant aspects to this approach. The functional knowledge that can be drawn from psychological research is a tool to identify neural mechanisms; the knowledge of the brain structure can guide the construction of far more sophisticated psychological models (Bechtel and Mundale, 1999). The index of free will that I am proposing (Lavazza and Inglese, 2015)—despite surely needing further refinement—might be useful to explore the brain mechanisms that underlie what appears in behavior as “free will”, which no longer seems to be an illusion, not even for neuroscientific research.

## Author Contributions

AL confirms being the sole contributor of this work and approved it for publication.

## Conflict of Interest Statement

The author declares that the research was conducted in the absence of any commercial or financial relationships that could be construed as a potential conflict of interest.
